# SARS-CoV-2 antibody seroprevalence in a large neuroimmunological patient cohort

**DOI:** 10.1007/s00415-021-10818-w

**Published:** 2021-10-05

**Authors:** Katharina Eisenhut, Stefan Buchka, Peter Eichhorn, Harald Meier, Fady Albashiti, Ulrich Mansmann, Miriam Schlüter, Joachim Havla, Tania Kümpfel

**Affiliations:** 1 Institute of Clinical Neuroimmunology, University Hospital, LMU Munich, Marchioninistr. 15, 81377 Munich, Germany; 2 Biomedical Center (BMC), Faculty of Medicine, LMU Munich, Martinsried, Germany; 3 Institute of Medical Information Processing, Biometry, and Epidemiology, Faculty of Medicine, LMU Munich, Munich, Germany; 4 Data Integration for Future Medicine (DIFUTURE) Consortium, LMU Munich, Munich, Germany; 5 Institute of Laboratory Medicine, University Hospital, LMU Munich, Munich, Germany; 6 Medical Technology and IT (MIT), University Hospital, LMU Munich, Munich, Germany

Dear Sirs,

The safety of patients with neuroimmunological conditions such as multiple sclerosis (MS) during the current SARS-CoV-2 pandemic has been a subject of major debate [1] and an accurate estimation of the burden of SARS-CoV-2 infection is pivotal. However, given the high prevalence of asymptomatic patients, the imperfect sensitivity of polymerase chain reaction (PCR) assays performed at a single time point, and limited testing capacity, the true number of SARS-CoV-2 infections likely exceeds the reported one. Serosurveys for SARS-CoV-2 antibodies (Abs) have so far only been reported for few neuroimmunological patient cohorts and often with only suboptimal assays [2, 3].

Here, we investigate the seroprevalence of SARS-CoV-2 Abs among neuroimmunology outpatient clinic patients from May 14th to September 30th, 2020, by two different assays and compare it to the seroprevalence of the general population. Further, precautionary health behavior was assessed to understand how possible over- or under-cautious demeanor may affect seroprevalence.

Of all patients who were admitted to the neuroimmunology outpatient clinic of LMU Hospital in Munich, Germany, during mentioned period, 509 gave written informed consent to participate in the study. A serum sample for SARS-CoV-2 antibody serology was taken from all included patients. Clinical data were retrospectively obtained using structured documentation of routine clinical data. Precautionary health behavior data was acquired from a digital analyzable, paper-based questionnaire (Online Resource 1) which was answered by 474 out of 509 included patients. In the questionnaire, adherence to official recommendations concerning curfew, hygiene recommendations, and reduction of social contacts during and after the lockdown in Germany in spring 2020 (calendar weeks 12–19) was enquired by symmetrical, balanced Likert scales ranging from 1 (“not at all”) to 5 (“very much”), respectively.

Each serum sample was tested for SARS-CoV-2 Ab by two assays at the Institute of Laboratory Medicine (LMU Hospital): Elecsys^®^ Anti-SARS-CoV-2 electrochemiluminescence immunoassay system measuring pan-Immunoglobulin (ECLIA, Roche-Diagnostics, Basel, Switzerland; hereafter Ro-pan-Ig) and Anti-SARS-CoV-2 enzyme-linked immunosorbent assay measuring IgG only (ELISA, EUROIMMUN, Lübeck, Germany; hereafter Eu-IgG) [4]. While Ro-pan-Ig only detects antibodies against the SARS-CoV-2 nucleocapsid antigen, Eu-IgG exclusively binds Abs to the receptor binding domain (RBD). According to the manufacturer, Ro-pan-Ig has 99.5% sensitivity and 99.8% specificity ≥ 14 days after a positive polymerase chain reaction (PCR) assay, respectively; the Eu-IgG sensitivity was 94.4% and specificity 99.6% [4].

Statistical analyses were performed using R-studio version 4.0.2 and WinBUGS (V 1.4.3, Imperial College and MRC, UK). To estimate the seroprevalence two different approaches were applied:

(i) For comparison with the seroprevalence of the general population in Munich, which was reported earlier using the Ro-pan-Ig assay only [5], Ro-pan-Ig seroprevalence in our cohort was adjusted for the manufacturer’s sensitivity and specificity. 95% confidence intervals (CI) were calculated by Poisson distribution.
(ii) Additionally, for a more robust estimation of SARS-CoV-2-Ab seroprevalence in our study population, a Bayesian approach was applied which considers both Ro-pan-Ig and Eu-IgG Ab assay results [6].

To compare two seroprevalences, a Chi^2^ test was applied. Questionnaire data were automatically retrieved by EvaSys software (ElectricPaper Evaluationssysteme, Lüneburg, Germany) and group comparisons were conducted by clustered Wilcoxon rank sum test.

Cohort details are provided in Table 1. In total, 11 out of 509 patients were identified as seropositive for SARS-CoV-2 Ab by either Ro-pan-Ig (*n* = 4) or Eu-IgG (*n* = 2) or both assays (*n* = 5), yielding an estimated seroprevalence of 1.27% (CI 0.46–2.44; approach (ii)) in our cumulated patient cohort (cumulated seroprevalence; cSP). The sensitivity- and specificity-adjusted cSP regarding Ro-pan-Ig only test results was slightly higher (1.58%; CI 0.49–2.67; approach (i)). The latter was compared to the SARS-CoV-2 Ab seroprevalence of the general population in Munich (1.82%; CI 1.28–2.37; Ro-pan-Ig), as obtained from the *Representative COVID-19 Cohort Munich* [5]. Even though a tendency can be observed with a slightly lower cSP in our patient cohort, this difference was not significant (Chi^2^ test, *p* = 0.96).

**Table 1:** Cohort overview

Characteristics	Patients *n* = 509
Age [years]	
< 18	1 (0.2%)
18–29	66 (13.0%)
30–39	113 (22.2%)
40–49	127 (25.0%)
50–59	127 (25.0%)
60–69	54 (10.6%)
≥ 70	21 (4.1%)
Sex	
F	343 (67.4%)
M	166 (32.6%)
Diagnosis	
Autoimmune encephalitis	16 (3.1%)
MS/CIS/RIS^a^	345 (67.7%)
Myasthenia gravis	23 (4.5%)
NMOSD	34 (6.7%)
Other neuroimmunological disease	91 (17.9%)
Immunotherapy	
Immunomodulatory^b^	26 (5.2%)
Immunosuppressive^c^	290 (58.0%)
B-cell-depleting therapy^d^	152 (30.4%)
None	184 (36.8%)
SARS-CoV-2	
History of (h/o) confirmed SARS-CoV-2 infection by PCR and/or serology^e^	3 (0.6%)
H/o symptomatic COVID- 19 disease^f^	2 (0.4%)
Positive SARS-CoV-2 Ab serology in our study^g^	11 (2.2%)
Estimated SARS-CoV-2 Ab seroprevalence (Ro-pan-Ig and Eu-IgG)^h^	1.27%; CI 0.46–2.44
Sensitivity- and specificity-adjusted SARS-CoV-2 Ab seroprevalence (Ro-pan-Ig)^i^	1.58%; CI 0.49–2.67

^a^EDSS mean: 3.1; range: 0–8.0

^b^Beta-interferon, Glatiramer acetate

^c^Anakinra, Azathioprine, Canakinumab, Cladribine, Dimethyl fumarate, Eculizumab, Fingolimod, Infliximab, intravenous immunoglobulins (IVIG), Mycophenolic acid (MFA), Methotrexate (MTX), Natalizumab, Ocrelizumab, oral steroids, Rituximab, Teriflunomide, Tocilizumab (in alphabetical order)

^d^Ocrelizumab, Rituximab. Only two patients on anti-CD20 therapy were seropositive for SARS-CoV-2 antibodies in our study and both infections were asymptomatic

^e^All patients with a h/o SARS-CoV-2 infection were tested positive for SARS-CoV-2 Ab in our study

^f^Of the two symptomatic patients, one was on immunotherapy with Anakinra, the other did not receive any immunotherapy

^g^Unadjusted seroprevalence considering patients positive for SARS-CoV-2 Ab in either Ro-pan-Ig or Eu-IgG or both assays

^h^See approach (ii)

^i^See approach (i)

Six of all 11 seropositive patients received immunotherapy at the time of serum sampling, among whom two were B-cell depleted due to anti-CD20 therapy. The sensitivity- and specificity-adjusted seroprevalence for Ro-pan-Ig of all patients receiving immunotherapy at the time of serum sampling (iSP) was 1.39% (CI 0.09–2.69; approach (i)). Compared to the SARS-CoV-2 Ab seroprevalence of the general population in Munich [5], iSP was lower, but again not significantly reduced (Chi^2^ test, *p* = 0.77).

All seropositive patients were either asymptomatic (81.8%; *n* = 9) or had a mild disease course (18.2%; *n* = 2), according to the WHO clinical progression scale for COVID-19 research (score < 4) [7]. A prior SARS-CoV-2 infection was previously unknown in 8 out of 11 SARS-CoV-2 Ab positive patients, yielding 72.7% incidental cases among all seropositives in our study population.

Our cohort largely adhered to official regulations both during and after the first lockdown in Germany (Fig. 1) without significant differences between participants with and without immunotherapy (clustered Wilcoxon rank sum test, *p* = 0.63). Of note, also all 11 SARS-CoV-2 Ab seropositive patients strictly adhered to recommendations as assessed in our study.

**Fig. 1:**
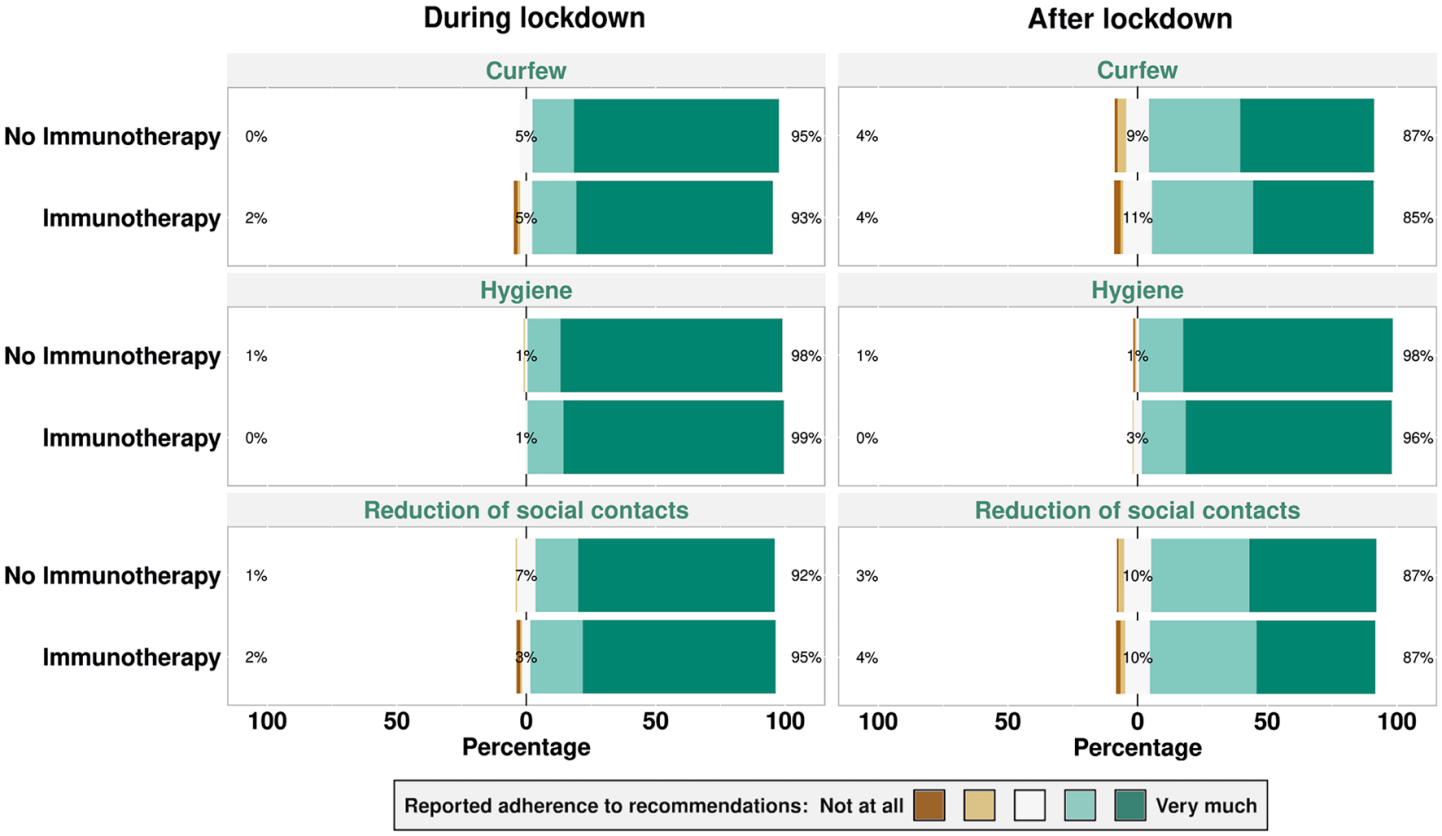
Self-reported precautionary health behavior of neuroimmunology outpatient clinic patients with and without immunotherapy during and after the first lockdown in Germany

Overall, the seroprevalence of SARS-CoV-2 Ab of neuroimmunological patients did not differ significantly from the seroprevalence of the general population. The high rate of incidental seropositives (72.7%) including two patients on anti-CD20 treatment further highlights that a serology study may be useful for estimating the true burden of SARS-CoV-2 infection also in patients undergoing immunotherapy. Nevertheless, false-positive Ab results, particularly due to cross-reaction with other endemic coronaviruses, should be considered [8]. Inter-assay discrepancies may be due to different test reactivities, as reported previously [4].

In accordance with previous data [9], precautionary health behavior recommendations were strictly adhered to in our whole cohort which may have contributed to the low number of infections in our vulnerable patient population. Also, self-reported behavior was independent of immunotherapy use, suggesting a general tendency toward cautious health behaviors during the current pandemic in our patient cohort.

This study has limitations. First, the *Representative Covid-19 Cohort Munich Study* used for reference was conducted from April to June 2020 and, thus, does not correspond exactly with the time period of our study. Comparison of seroprevalence is further limited due to different cutoffs for the Ro-pan-Ig assay (0.4 in the *Representative Covid-19 Cohort Munich Study* instead of 1.0 as intended by the manufacturer and as applied in this study). Also, seroprevalence in mentioned study may have been underestimated since they exclusively relied on Ro-pan-Ig assay results in the final analysis. Furthermore, in our study, precautionary health behavior was self-reported and appropriate controls are lacking, restricting any direct causal link between behavior and seroprevalence. Generally, the presented data are limited to a low-prevalence setting. Nonetheless, this study emphasizes the importance of serosurveys as a public health practice to avoid underestimation of SARS-CoV-2 infection burden in patients with neuroimmunological disease.

## Supplementary Information

Below is the link to the electronic supplementary material.

Supplementary file1 Online Resource 1: Questionnaire on precautionary health behavior. (PDF 407 KB)

## Data Availability

The datasets generated and analyzed during the study are available from the corresponding author on reasonable request.

## References

[1] Sharifian-Dorche M, Sahraian MA, Fadda G (2021). COVID-19 and disease-modifying therapies in patients with demyelinating diseases of the central nervous system: a systematic review. Mult Scler Relat Disord.

[2] Capasso N, Palladino R, Montella E (2020). Prevalence of SARS-CoV-2 antibodies in multiple sclerosis: the hidden part of the iceberg. J Clin Med.

[3] van Kempen ZLE, Strijbis EMM, Al MMCT (2021). SARS-CoV-2 antibodies in adult patients with multiple sclerosis in the Amsterdam MS Cohort. JAMA Neurol.

[4] Zilla M, Wheeler BJ, Keetch C (2021). Variable performance in 6 commercial SARS-CoV-2 antibody assays may affect convalescent plasma and seroprevalence screening. Am J Clin Pathol.

[5] Pritsch M, Radon K, Bakuli A (2021). Prevalence and risk factors of infection in the representative COVID-19 cohort Munich. Int J Environ Res Public Health.

[6] Joseph L, Gyorkos TW, Coupal L (1995). Bayesian estimation of disease prevalence and the parameters of diagnostic tests in the absence of a gold standard. Am J Epidemiol.

[7] WHO Working Group on the Clinical Characterisation and Management of COVID-19 infection (2020). A minimal common outcome measure set for COVID-19 clinical research. Lancet Infect Dis.

[8] Okba NMA, Müller MA, Li W (2020). Severe acute respiratory syndrome Coronavirus 2-specific antibody responses in coronavirus disease patients. Emerg Infect Dis.

[9] Alnajashi H, Jabbad R (2020). Behavioral practices of patients with multiple sclerosis during Covid-19 pandemic. PLoS ONE.

