# Supplementary material for: SARS-CoV-2 antibody seroprevalence in a large neuroimmunological patient cohort
Source: J Neurol. 2021 Oct 5;269(3):1133–7. doi: 10.1007/s00415-021-10818-w (PMC8491170; doi:10.1007/s00415-021-10818-w)
Supplement: Supplementary file 1 — Supplementary file1 Online Resource 1: Questionnaire on precautionary health behavior. (PDF 407 KB) [file 415_2021_10818_MOESM1_ESM.pdf]

Institute of Clinical Neuroimmunology
LMU University Hospital

COVID-19 and Neuroimmunology
Questionnaire on precautionary health behavior
during the SARS-CoV-2 pandemic

Bitte so markieren: ☐ ☒ ☐ ☐ ☐ Bitte verwenden Sie einen Kugelschreiber oder nicht zu starken Filzstift. Dieser Fragebogen wird maschinell erfasst.
Korrektur: ☐ ■ ☐ ☒ ☐ Bitte beachten Sie im Interesse einer optimalen Datenerfassung die links gegebenen Hinweise beim Ausfüllen.

## 1. **During the "lockwdown"**, how strongly did you adhere to official recommendations regarding the following?

| | | | | | | | |
|---|---|---|---|---|---|---|---|
| 1.1 Curfew | Not at all | ☐ | ☐ | ☐ | ☐ | ☐ | Very much |
| 1.2 Hygiene | Not at all | ☐ | ☐ | ☐ | ☐ | ☐ | Very much |
| 1.3 Reduction of social contacts | Not at all | ☐ | ☐ | ☐ | ☐ | ☐ | Very much |

## 2. **After the "lockdown"**, how strongly did you adhere to official recommendation regarding the following?

| | | | | | | | |
|---|---|---|---|---|---|---|---|
| 2.1 Curfew | Not at all | ☐ | ☐ | ☐ | ☐ | ☐ | Very much |
| 2.2 Hygiene | Not at all | ☐ | ☐ | ☐ | ☐ | ☐ | Very much |
| 2.3 Reduction of social contacts | Not at all | ☐ | ☐ | ☐ | ☐ | ☐ | Very much |

## 3. To be filled in by the study team

BiobaMS ID

| | | | | |
|---|---|---|---|---|
| 1 | ☐ | ☐ | ☐ | ☐ |
| 2 | ☐ | ☐ | ☐ | ☐ |
| 3 | ☐ | ☐ | ☐ | ☐ |
| 4 | ☐ | ☐ | ☐ | ☐ |
| 5 | ☐ | ☐ | ☐ | ☐ |
| 6 | ☐ | ☐ | ☐ | ☐ |
| 7 | ☐ | ☐ | ☐ | ☐ |
| 8 | ☐ | ☐ | ☐ | ☐ |
| 9 | ☐ | ☐ | ☐ | ☐ |
| 0 | ☐ | ☐ | ☐ | ☐ |

SARS-CoV-2 antibody seroprevalence and precautionary health behavior in a large neuroimmunological patient cohort; Journal of Neurology; Eisenhut et al.; Corresponding author:
Prof. Tania Kümpfel, tania.kuempfel@med.uni-muenchen.de
